# Evaluation of the Mean Cost and Activity Based Cost in the Diagnosis of Pulmonary Tuberculosis in the Laboratory Routine of a High-Complexity Hospital in Brazil

**DOI:** 10.3389/fmicb.2017.00249

**Published:** 2017-02-15

**Authors:** Isabela N. de Almeida, Lida J. de Assis Figueredo, Valéria M. Soares, Maria C. Vater, Suely Alves, Wânia da Silva Carvalho, Afrânio L. Kritski, Silvana S. de Miranda

**Affiliations:** ^1^Research Laboratory in Mycobacteria, School of Medicine of the Federal University of Minas GeraisBelo Horizonte, Brazil; ^2^Research Center for Tuberculosis, Academic Program in Tuberculosis, School of Medicine of the Federal University of Rio de JaneiroRio de Janeiro, Brazil; ^3^School of Pharmacy of the Federal University of Minas GeraisBelo Horizonte, Brazil

**Keywords:** tuberculosis, health system, cost analysis, mycobacteria, technology

## Abstract

At a global level, with the increase in healthcare costs, there is a need to assess the economic impact of the incorporation of new technologies in different health disorders in different countries. There is scarce information regarding costs incurred with the use of current or new diagnostic tests for tuberculosis or from the vantage point of their incorporation within the healthcare systems of high-burden countries. The present study aimed to assess the mean cost and the activity based cost of the laboratory diagnosis for tuberculosis by means of conventional techniques and from the Detect TB^®^LabTest molecular test kit in a general high-complexity hospital of the public health system in Brazil. Cost analysis was performed by means of primary data, collected in the Mycobacteria and Molecular Biology Laboratory in 2013. The mean cost and activity based cost were, respectively, U$10.06/U$5.61 for centrifuged bacilloscopy by Ziehl Neelsen (ZN) and Auramine (AU); U$7.42/U$4.15 for direct bacilloscopy by ZN; U$27.38/U$16.50 for culture in a Loweinstein-Jensen solid medium; and U$115.74/U$73.46 for the Detect TB^®^LabTest Kit. The calculation of the ABC should be used in making decisions by administrators to be the best method of assessing the costs of conventional techniques and molecular method for providing the real value of the tests. So it is need to calculate the ABC, and not of the mean cost, in various scenarios before incorporating new technologies in health institutions.

## Introduction

Tuberculosis (TB) remains as one of the central problems of world public health. In 2015, in Brazil, some 71,221 new cases were notified, with 73% confirmed by laboratory diagnosis (bacilloscopy or culture), and 71% presented treatment success (WHO, 2016). The diagnosis of TB by means of high-quality mycobacteriological exams associated with clinical/epidemiological and radiological criteria represent efficient strategies to TB control (BRASIL, 2010). However, these conventional methods present some disadvantages, such as low sensitivity and specificity, as in the case of the bacilloscopy and chest X-ray (The International Journal of Tuberculosis, and Lung Disease [IJTLD], 2012).

The culture, considered the “gold standard”, has limitations, which are inherently linked to the time needed to obtain the results and the need for a robust infrastructure in the laboratory, which is limited to reference centers (Gholoobi et al., 2014). Molecular diagnosis is reported to have higher sensitivity than bacilloscopy and faster than cultures (Almeida et al., 2015). As of 2008, the World Health Organization (WHO) endorsed the use of molecular methods to detect TB and drug-resistant TB as an alternative to a faster diagnosis of TB (WHO, 2008).

These methods present a high specificity, though lower sensitivity in patients with negative bacilloscopy results. The most commercial molecular methods used to detect TB and drug-resistant TB are the GenoType^®^MTBDRplus (Biomeuriéx) and the Xpert MTB/RIF^®^ (Cepheid). One major advantage of these methods is that these can provide results more quickly than the culture, yet the greatest disadvantage is that they are too expensive and too complex for routine use in contexts with limited resources (Steingart et al., 2015). In Brazil, the Xpert MTB/RIF^®^ was introduced into the public system by recommendation from the Ministry of Health in 2014. A new kit developed in Brazil (Detect TB^®^LabTestKit, MG, Brazil) has been used for the molecular diagnoses of the *Mycobacterium tuberculosis* Complex (MTBC) (BRASIL, 2011; Michelon et al., 2011; Schimd et al., 2014).

Nevertheless, in high-burden countries, data concerning the costs incurred with the use of diagnostic tests for TB are scarce in routine conditions, and the TB control programs require decision-making based on algorithms of diagnoses that consider the laboratory costs, as well as those related to clinical treatment and the local prevalence of TB (Shah et al., 2013). At the global level, with the increase in healthcare costs, there is a growing need to assess TB diagnostic methods, both current methods and those planned for future implementation in different countries (Silva, 2003; BRASIL, 2011).

While there are large numbers of studies on accuracy of TB diagnostic tests, there are few studies that are focused on cost. Study conducted in Thailand demonstrated that the costs by Ziehl Neelsen (ZN) and Auramine (AU) were very close. In Zambia compared the values of the culture by different techniques in liquid and solid media found few variations. In South Africa, the cost of molecular method (Xpert MTB/RIF^®^) was similar to conventional automated liquid culture-based methods and the GenoType^®^MTBDRplus was higher (Mueller et al., 2008; Sohn et al., 2009a,b; Shah et al., 2013).

Within the methodologies used to calculate the costs of health services, activity based costing (ABC) is appropriate for complex organizations, in which the products consume resources in a highly heterogeneous manner, such as occurs in hospitals. The benefits of ABC are many, especially because it improves managerial decisions; facilitates the determination of relevant costs; allows for the identification of actions geared toward the reduction of overhead costs; provides a greater precision in product costs; determines the costs of services/products; offers support in the negotiation of contracts; provides support in the increase in revenue, helping customers to understand the cost reductions as consequence of the use of their products and services; gives support for benchmarking; and determines the remainder of shared services (BRASIL, 2006).

A study was recently conducted in Brazil aimed at supplying subsidies for managers to identify the main cost guidelines and possible gains in efficiency and effectiveness when adopting the XpertMTB/RIF, using the ABC as a cost methodology to pinpoint the advantage of being able to observe a significant quantity of tests, thus making it possible to identify a standard cost and conduct a detailed inventory of the cost items (Pinto et al., 2015).

In the Brazilian context, considering that the majority of reference laboratories are structured to conduct bacilloscopies and cultures to detect mycobacteria, it thus becomes necessary to measure the real costs of both these technologies and the Detect TB^®^ kit, the only national molecular method (BRASIL, 2010; Michelon et al., 2011).

Therefore, the present study aimed to assess the mean cost and ABC of the laboratory diagnosis of TB by means of conventional techniques and the Detect TB^®^LabTest molecular test kit in a high-complexity general hospital from the public health system.

## Materials and Methods

### Design and Study Site

This study’s cost analysis was performed by means of primary data collected in the Research Laboratory in Mycobacteria (RLM) of the Federal University of Minas Gerais (FUMG) School of Medicine (SM), as well as in the Molecular Biology and Public Health Laboratory (MBPH) of the FUMG School of Pharmacy (SP), from January to December 2013. At RLM, TB laboratory diagnoses are conducted on patients who receive medical care at the hospital complex of the Clinical Hospital (CH)/FUMG, a public and general university hospital that conducts educational, research, and medical care activities. CH/FUMG is a reference hospital in the municipal and state health system concerning medical care provided to patients with mid- and high-complexity pathologies, and consists of one hospital unit and seven outpatient care centers (UFMG, 2015).

### Study’s Cost Analysis

The costs of TB laboratory diagnoses (bacilloscopy and culture) were evaluated based on two methodologies: by mean cost and by activity. Mean Cost is calculated through the total cost divided by the quantity produced over a determined period of time (BRASIL, 2006), which, in this study, considered the quantity of exams conducted per month.

**FIGURE 1:**
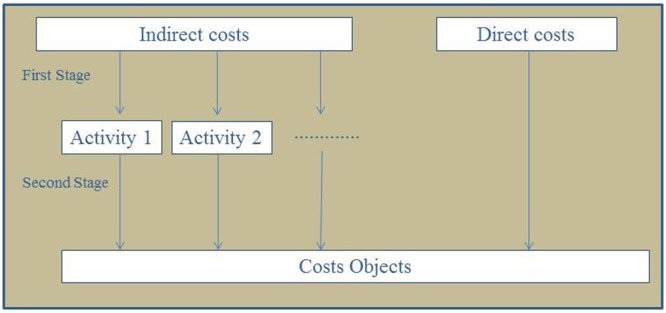
**Cost flow in the activity based costing systems.** ^∗^Activity = performing one diagnostic test (bacilloscopy, culture, or Detect TB^®^).

The ABC is calculated considering an activity as the denominator for the calculation of the unit cost per activity, rather than the real consumed quantity. The aim of this procedure is to avoid fluctuations in the calculation of the activity’s unit cost based on the variation of the real processed quantity. The basic principle of this system is to make the largest possible number of proportional and non-proportional costs more direct, through cost guidelines, as illustrated in **Figure 1** (Khoury and Ancelevicz, 2000).

To calculate both costs (mean/ABC), this study verified the values of all cost components of the assessed diagnostic methods, such as: infrastructure, equipment, inputs, personal protective equipment (PPE), human resources, and the maintenance of biosafety laboratories (B3), according to the daily routine at RLM/SM/FUMG in 2013. These data were collected by consulting the purchasing, human resources, and maintenance sectors after prior institutional authorization and study approval by the Ethics Committee (CAAE -11821913.6.000.5257, CAAE – 0223.2412.7.1001.5149, DEPE/CH, protocol number 139/12).

### Diagnostic Tests of Tuberculosis Laboratory Routine

The RLM/SM/FUMG counts on two work flows to perform a bacilloscopy: (1) direct bacilloscopy by ZN in samples from emergency rooms and wards at the FUMG Clinical Hospital for the release of exam results in up to four hours; (2) centrifuged bacilloscopy to execute staining by means of the ZN method and fluorescent (AU) of all of the other samples that arrive from the Hospital Complex (out-patient care), including the samples in which the direct bacilloscopy was performed.

The culture is performed in a Loweinstein-Jensen (LJ) solid medium, using four tubes –two tubes without drugs and two with *p*-Nitrobenzoic acid (PNB) and 2-thyophenecarboxylic acid (TCH). The samples are decontaminated by the *N*-Acetyl L-Cysteine (NALC) method (WHO, 2009). Monthly, the RLM conducts an average of 100 bascilloscopies and 91 respiratory sample cultures, such as sputum, bronchoalveolar lavage, and endotracheal aspirates, as well as of extra-pulmonary samples, such as cerebrospinal fluid, urine, biopsies, among others.

To calculate the costs of the Detect TB^®^ kit, only the respiratory samples were considered, following both the manufacturer’s recommendations (Labtest, 2012) and all of the conditions established by RLM/SM/FUMG and MBPH/SP/FUMG. The number of samples included in the calculation for the Detect TB^®^ kit was only one per patient. All costs were expressed in U$, using an exchange rate of US$ 1 = R$ 2,34 in 2013 according Brazilian Central Bank.

## Results

### The Cost of Infrastructure

The results of the mean cost of the RLM/SM/FUMG infrastructure are shown in **Table 1**; these items did not influence the ABC of the verified methods. It can be observed that security is the item that most influenced the mean cost.

**Table 1:** Cost of Infrastructure of Research Laboratory in Mycobacteria and Molecular Biology and Public Health Laboratory, FUMG.

Item	Total monthly value	Mean cost
Telephone	U$ 227.35	U$ 0.45
Electricity	U$ 291.02	U$ 0.58
Water	U$ 400.85	U$ 0.80
Security	U$ 3,571.52	U$ 7.14
Maintenance of B3	U$ 890.17	U$ 1.78

### The Cost of Diagnostic Tests of Tuberculosis Laboratory Routine

The main items that influenced the mean costs and ABC of the bacilloscopy, culture, and Detect TB^®^ kit are shown in **Tables 2–4**, where one can observe an individual difference in the mean cost of the equipment and a similarity of ABC for inputs. The unit value of the disposable apron observed in **Tables 2 and 3** is higher than the other inputs, but did not affect the increase in mean cost and ABC. The mean cost and ABC of the assessed technologies are shown in **Table 4**; the cost components of each exam are listed in **Table 5**. The Detect TB^®^ kit presented the highest cost, followed by the culture and the centrifuged and direct bacilloscopies. Among the cost components, in all of the assessed methods, the inputs were what most influenced the increase in the final value of each test followed by human resources, equipment and permanent materials (**Table 6**).

**Table 2:** Solid culture on Loweinsten–Jensen – main cost elements

Equipment	Quantity of laboratories	Unit value	Mean cost	Activity based cost
Refrigerated centrifuge	1	U$ 8,588.03	U$ 0.38	U$ 0.05
Biological Safety Cabinet	2	U$ 38,461.53	U$ 3.52	U$ 0.48
Refrigerator	2	U$ 512.82	–	–
Freezer –20°C.	1	U$ 769.23	–	–
Bacteriological incubator	3	U$ 1,945.72	–	U$ 0.03

**Inputs**	**Quantity per exam**	**Unit value**	**Mean cost**	**Activity based cost**

Polypropylene centrifuge tubes	1	U$ 0.51	U$ 0.55	U$ 0.51
Disposable apron (unit)	1^∗^	U$ 7.99	U$ 1.46	U$ 1.33
Tube with LJ medium	2	U$ 1.98	U$ 3.97	U$ 3.97
Tube with LJ medium with TCH	1	U$ 1.98	U$ 1.98	U$ 1.98
Tube with LJ-PNB medium	1	U$ 1.98	U$ 1.98	U$ 1.98

*^∗^1 per day of work; LJ, Loweinstein–Jensen; TCH, 2-thyophenecarboxylic acid; PNB, *p*-Nitrobenzoic acid. Mean Cost is calculated through the total cost divided by the quantity produced over a determined period of time (BRASIL, 2006). ABC is calculated considering an activity as the denominator for the calculation of the unit cost per activity, rather than the real consumed quantity (Khoury and Ancelevicz, 2000). Exchange rate of US$ 1 = R$ 2,34 in 2013 according Brazilian Central Bank.*

**Table 3:** Cost of the main equipment and inputs for bacilloscopy.

Equipment	Quantity of laboratories	Unit value	Mean cost	Activity based cost
Test stand with running water	1	U$ 2,564.10	–	U$ 0.01
Binocular microscope	1	U$ 1,227.35	–	–
Led microscope	1	U$ 24,798.88	U$ 1.02	U$ 0.15
Refrigerated centrifuge	1	U$ 8,588.03	–	U$ 0.05
Biological Safety Cabinet	2	U$ 38,461.53	U$ 3.20	U$ 0.48
Refrigerator	2	U$ 512.82	–	–
Freezer –20°C	1	U$ 769.23	–	–

**Inputs**	**Quantity per exam**	**Unit value**	**Mean cost**	**Activity based cost**

Glass slides for microscopy	2	U$ 0.62	U$ 1.24	U$ 1.24
Polypropylene centrifuge tubes	1	U$ 0.51	U$ 0.51	U$ 0.51
Disposable apron (unit)	1^∗^	U$ 7.99	U$ 1.33	U$ 1.33

*^∗^1 per day of work. Mean Cost is calculated through the total cost divided by the quantity produced over a determined period of time (BRASIL, 2006). ABC is calculated considering an activity as the denominator for the calculation of the unit cost per activity, rather than the real consumed quantity (Khoury and Ancelevicz, 2000). Exchange rate of US$ 1 = R$ 2,34 in 2013 according Brazilian Central Bank.*

**Table 4:** Main laboratory issued with impact on Detect TB^®^ test costs.

Equipment	Quantity of laboratories	Unit value	Mean cost	Activity based cost
Biological Safety Cabinet	2	U$ 38,461.53	U$ 8.01	U$ 4.45
MiliQ Water Purifier	2	U$ 7,692.30	U$ 1.60	U$ 0.88
Elisa Scanner	1	U$11,538.46	U$ 1.19	U$ 0.66
Hybridization Incubator	1	U$ 5,769.23	U$ 0.59	U$ 0.33
Thermocycler	1	U$10,256.41	U$ 1.06	U$ 0.59

**Inputs**	**Quantity per exam**	**Unit value**	**Mean cost**	**Activity based cost**

Detect TB^®^ KIT	–	U$ 2,289.74	U$ 31.80	U$ 31.80
Disposable apron (unit)	1^∗^	U$ 7.99	U$ 1.33	U$ 1.33
Nozzles with filter (26 per exam)	26	U$ 0.09	U$ 2.55	U$ 2.55
Microtubes	4	–	–	U$ 0.03
Disposable gloves	1	U$ 0.05	–	U$ 0.11

*^∗^1 per day of work. Mean Cost is calculated through the total cost divided by the quantity produced over a determined period of time (BRASIL, 2006). ABC is calculated considering an activity as the denominator for the calculation of the unit cost per activity, rather than the real consumed quantity (Khoury and Ancelevicz, 2000). Exchange rate of US$ 1 = R$ 2,34 in 2013 according Brazilian Central Bank.*

**Table 5:** Mean and activity based cost from the Research Laboratory in Mycobacteria and Molecular Biology and Public Health Laboratory, FUMG.

Method	Number of samples/month	Mean cost	Activity based cost
Centrifuged Bacilloscopy (ZN and AU)	100	U$ 10.06	U$ 5.61
Centrifuged Bacilloscopy (AU)	100	U$ 9.26	U$ 4.85
Centrifuged Bacilloscopy (ZN)	100	U$ 8.29	U$ 4.72
Direct Bacilloscopy (ZN) ^∗^	100	U$ 7.42	U$ 4.15
Culture in LJ Solid Medium	91	U$ 27.38	U$ 16.50
Detect TB^®^	40	U$ 115.74	U$ 73.46

*^∗^Performed when request for bacilloscopy is urgent. ZN, Ziehl Neelsen, AU, Auramine O. ABC is calculated considering an activity as the denominator for the calculation of the unit cost per activity, rather than the real consumed quantity (Khoury and Ancelevicz, 2000).*

*Exchange rate of US$ 1 = R$ 2,34 in 2013 according Brazilian Central Bank.*

**Table 6:** Cost components of activity based cost for each diagnostic test.

	Inputs per test	Equipment and permanent materials	Human resources	Total
Detect TB^®^	U$ 36.46 (55%)	U$ 7.62 (11%)	U$ 29.35 (34%)	U$ 73.46
Solid Medium Culture	U$ 11.40 (78%)	U$ 0.62 (4%)	U$ 3.19 (18%)	U$ 16.50
Bacilloscopy by Flourescence (AU)	U$ 3.08 (64%)	U$ 0.50 (10%)	U$ 1.26 (26%)	U$ 4.85
Centrifuged Bacilloscopy (ZN)	U$ 3.10 (66%)	U$ 0.35 (8%)	U$ 1.26 (26%)	U$ 4.72
Direct Bacilloscopy (ZN)	U$ 2.56 (62%)	U$ 0.30 (7%)	U$ 1.28 (31%)	U$ 4.15
Centrifuged Bacilloscopy (ZN and AU)	U$ 3.84 (69%)	U$ 0.50 (9%)	U$ 1.26 (22%)	U$ 5.61

*ZN, Ziehl Neelsen; AU, Auramine O. ABC is calculated considering an activity as the denominator for the calculation of the unit cost per activity, rather than the real consumed quantity (Khoury and Ancelevicz, 2000).*

*Exchange rate of US$ 1 = R$ 2,34 in 2013 according Brazilian Central Bank.*

## Discussion

The mean cost was higher than the ABC in the diagnosis of pulmonary tuberculosis in the laboratory routine of a High-Complexity Hospital in Brazil. The results of the mean cost and ABC found in this study were of utmost importance for the re-structuring of laboratory activities, since, faced with these results, the routine of performing one slide stained with ZN and one with AU in centrifuged bacilloscopy was changed to only one slide stained with AU.

The impact that this result caused in the laboratory’s routine showed the substantial advantage of having used the ABC as the methodology, as well as the importance of conducting cost studies based on data that has been duly computed and not merely estimated based on other studies or the price of the Unique Health System in Brazil. These data can serve as a parameter for other public laboratories in Brazil that perform the same TB diagnostic methods, in addition to affirming that the correct count of the costs incurred in public services is an important instrument for social control and the assessment of the efficiency of services rendered (Martins, 2003).

The Detect TB^®^ kit was the method that presented the highest costs. Nonetheless, it cannot be inferred whether or not the implementation of this would in fact be more costly, given that the sensitivity and specificity described by Michelon et al. (2011), showed from 75 to 100% and 98 to 100%, respectively, as compared to the bacilloscopy and culture, and presents the advantage of a shorter waiting time for the release of the result as compared to the culture. This fact reinforces the need for a cost study on the effectiveness of this test in low-prevalence locations, such as that carried out in the present work.

The values found for the Detect TB^®^ kit are similar to other studies where molecular tests are assessed together with conventional techniques (Shah et al., 2013). The value of the Detect TB^®^ kit (U$73.46) found in our study is less than the value of the Xpert MTB/RIF^®^ molecular method (U$ 503.05, value of the cartridge) when not subsidized by the health system (BRASIL, 2013; FIND, 2013).

As regards, the bacilloscopy, it could be observed that there is no need to perform two methods of staining, since the costs are approximate and the AU technique is quick and sensitive, and does not expose the technicians to toxic vapors released when heating ZN (BRASIL, 2010). Despite the lower cost of staining by ZN without centrifugation, this method is less sensitive than centrifugation (WHO, 2009). The values of bacilloscopy found in the present study differ from the values cited in a similar study carried out in South Africa, where the ABC of the bacilloscopy stained with AU presented the value of U$3.40 and with ZN of U$2.25. Nevertheless, the values from the present study are near those found in different scenarios in Brazil, where the ABC of both staining methods was U$4.86 and for the mean cost was U$ 6.05 (BRASIL, 2013; Shah et al., 2013). The costs of bacilloscopy in Thailand by ZN and AU were US$1.16 and US$1.03 respectively. In Zambia compared the values of the culture by means of different techniques in liquid and solid media found a variation between US$28 and $32. However, these studies did not evaluate the mean cost and ABC. (Sohn et al., 2009a,b).

In the case of the culture, costs identified in the present study (U$ 16.50) were similar to other studies carried out in Brazil and Africa, with a variation from U$ 12.35 to U$ 28.00 (Mueller et al., 2008; Chihota et al., 2010; BRASIL, 2013).

The results of the ABC of the bacilloscopy and culture found in this study and those reported in national literature (BRASIL, 2013) are higher than the Brazilian Unified Health System’s pay (U$ 1.79 for bacilloscopy and U$ 2.29 for cultures), which alerts to the fact that these values do not reflect the real cost. This divergence confirms the relevance of the assessment of ABC, as the horizontal view of this parameter made it possible to reach an analysis that was not restricted to profit. This view is based on the planning, performing, and aid in strategic decision-making, as well as in the changes in processes, the elimination of waste, and the drafting of estimates based on the executed activities, thus increasing the efficiency of public services (Martins, 2003; Alonso, 2009).

In some countries of Asia and America the health system is private or public–private. In this context, the cost studies are important to improve the ability of management and financing of TB control programs (WHO, 2016).

The limitation of this study, rely on the fact that the individual costs of the patients were not inferred, such as: transport, outcome, among others, which are important variables for future studies on cost effectiveness and the implementation of an algorithm in laboratory diagnoses and strategies for the TB control. In addition, this study was carried out with local data from an NB3 laboratory geared exclusively towards the diagnosis of TB and other mycobacteria from high-complexity hospitals and from Molecular Biology laboratories.

## Conclusion

The calculation of the ABC should be used in making decisions by administrators to be the best method of assessing the costs of conventional techniques and molecular method for providing the real value of the tests. So, it is need to calculate the ABC, and not of the mean cost, in various scenarios before incorporating new technologies in health institutions.

## Author Contributions

Design of study: IA and SdM. Development of Cost chain: MV and SA. Data analysis: IA, MV, and SA. Article writing: IA, VS, WdSC, LJdAF, and SdM. Article review: MV, SA, AK. Production of the article’s final version: IA, VS, LJdAF, WdSC, MV, SA, AK. Promotion of study financing: SdM and AK. All the authors read and approved the final manuscript.

## Conflict of Interest Statement

The authors declare that the research was conducted in the absence of any commercial or financial relationships that could be construed as a potential conflict of interest.
